# St. Bartholomew’s Hospital

**Published:** 1852-11

**Authors:** 


The medical schools of London are dependent on and connected with the hospitals. The following list of the physicians and faculties in these joint institutions, we condense from the London Lancet for September 25th.

ST. BARTHOLOMEW'S HOSPITAL.—*Physicians*—Drs. Hue, Roupell, and Burrows. *Surgeons*—Messrs. Lawrence, Stanley, and Lloyd. *Assistant Physicians*—Drs. Farre, Jeaffreson, and Black. *Assistant Surgeons*—Messrs. Skey, Wormald, and Paget. *Physician Accoucheur*—Dr. West.

ST. BARTHOLOMEW'S COLLEGE. *Winter Session.*—Medicine, Dr. Burrows. Descriptive and Surgical Anatomy, Mr. Skey. General and Morbid Anatomy, and Physiology, Mr. Paget. Practical Anatomy, Messrs. Holden, Holmes, and Coote. Surgery, Mr. Lawrence. Chemistry, Mr. Stenhouse. *Summer Session.*—Materia Medica and Therapeutics, Dr. Roupell. Midwifery and the Diseases of Women and Children, Dr. West. Botany, Dr. Farre. Practical Chemistry, Mr. Stenhouse. Forensic Medicine, Dr. Baly. Comparative Anatomy, Mr. McWhinnie. Natural Philosophy, Dr. Gibbon.

